# Time to first cardiovascular hospitalization after guideline-based treatment optimization: A multicenter retrospective cohort study in northwest Ethiopia

**DOI:** 10.1016/j.ijcrp.2026.200607

**Published:** 2026-02-17

**Authors:** Getachew Yitayew Tarekegn, Abel Temeche Kassaw, Tilaye Arega Moges, Samuel Agegnew Wondm, Samuel Berihun Dagnew, Tigabu Eskeziya Zerihun, Desalegn Addis Mussie, Fasil Bayafers Tamene, Abaynesh Fentahun Bekalu, Woretaw Sisay zewdu

**Affiliations:** a Department of Pharmacy, College of Health Science, Debre Tabor University, Debre Tabor, Ethiopia; b Department of Pharmacy, College of Health Science, Debre Markos University, Debre Markos, Ethiopia; c Department of Clinical Pharmacy, Pharmacy Education and Clinical Service Directorate, Debre Tabor University, Debre Tabor, Ethiopia; d Department of Clinical Pharmacy, School of Pharmacy, College of Medicine and Health Science, University of Gondar, Gondar, Ethiopia

**Keywords:** Cardiovascular diseases, Hospitalization, Predictors, Ethiopia, Guideline-directed therapy, Comorbidity

## Abstract

**Background:**

Cardiovascular hospitalization is a leading cause of morbidity and mortality, especially in low-resource settings. Evidence on predictors in Ethiopia is limited, hindering targeted interventions.

**Objective:**

To assess the incidence and predictors of first cardiovascular hospitalization within one year of treatment optimization in Northwest Ethiopia.

**Methods:**

A retrospective cohort study was conducted at three tertiary hospitals. Baseline demographic, clinical, and treatment data were extracted from medical records of patients receiving optimized cardiovascular therapy. Multivariate Cox proportional hazards regression with robust standard errors identified independent predictors of first hospitalization.

**Results:**

Among 470 patients, 218 (46.4%) were hospitalized within 12 months; median time to first admission was 100 days (IQR 55–160). Independent predictors of shorter hospitalization-free survival included partial or no guideline-directed medical therapy (HR 1.71, 95% CI: 1.30–2.25), Charlson Comorbidity Index ≥3 (HR 1.62, 95% CI: 1.25–2.10), heart failure with reduced ejection fraction (HR 1.46, 95% CI: 1.10–1.94), chronic kidney disease (HR 1.41, 95% CI: 1.02–1.95), current smoking (HR 1.40, 95% CI: 1.01–1.95), low physical activity (HR 1.35, 95% CI: 1.03–1.77), age ≥65 years (HR 1.36, 95% CI: 1.05–1.76), and absence of a scheduled follow-up plan (HR 1.60, 95% CI: 1.18–2.16).

**Conclusions:**

Nearly half of patients experienced hospitalization within one year, mostly within 3–4 months. Ensuring guideline-directed therapy, structured follow-up, and addressing modifiable risk factors may reduce early cardiovascular admissions in low-resource settings.

## 1 Introduction

Cardiovascular diseases (CVDs) remain the leading cause of global morbidity and mortality, accounting for nearly 18 million deaths annually, representing about one-third of all deaths worldwide [1]. The burden of CVD is disproportionately high in low- and middle-income countries (LMICs), where over 75% of CVD-related deaths occur [2]. In Ethiopia, CVDs account for approximately 20% of non-communicable disease deaths, reflecting an escalating public health challenge driven by urbanization, demographic shifts, and rising prevalence of risk factors such as hypertension, diabetes, obesity, and tobacco use [3].

Hospitalization for cardiovascular causes is a critical marker of disease progression and an important contributor to increased morbidities and mortality and healthcare costs. Global rehospitalization rates for patients with CVD range from 25% to 40% within 1 year, with higher rates reported in the resource-linting setting [4]. Despite the vulnerability of patients following treatment optimization, data on the incidence and timing of the first cardiovascular hospitalization and its predictors in Ethiopia and similar LMIC contexts remain limited.

This lack of data is compounded by fragmented health information systems, small single-center studies, and inconsistent reporting, all of which limit the understanding of hospitalization patterns and hinder the development of effective interventions [5]. Suboptimal adherence to guideline-directed medical therapy, limited access to advanced treatments, and socioeconomic barriers further increase three hospitalization risk factors but are poor characteristics in these settings [[6], [7], [8], [9]].

Treatment optimization, defined as initiating or adjusting cardiovascular therapies, including both pharmacologic and non-pharmacologic interventions, in accordance with current clinical guidelines, is essential to achieving optimal patient outcomes. Adherence to GDMT is a key determinant in reducing cardiovascular hospitalization risk [10,11].

Accordingly, this study aimed to determine the incidence and predictors of first cardiovascular hospitalization within 12 months after treatment optimization among patients attending tertiary hospitals in Northwest Ethiopia. The findings are intended to inform clinical practice and public health strategies to reduce the burden of cardiovascular disease in resource-constrained settings.

## 2 Methods

### 2.1 Study design and setting

A retrospective cohort study was conducted at three tertiary hospitals in Northwest Ethiopia, providing comprehensive cardiovascular care to patients from urban and rural areas from January to December 2023.

### 2.2 Study population

The study included adult patients (≥18 years) with a confirmed diagnosis of cardiovascular disease who were initiating therapy optimization according to current guideline recommendations, including but not limited to guideline-directed medical therapy (GDMT). Patients were recruited from three tertiary hospitals, regardless of whether therapy was started during hospitalization or after discharge in the outpatient setting, to ensure a representative sample of real-world cardiovascular care. Patients with incomplete medical records or terminal illnesses were excluded to maintain a reliable assessment of therapy initiation. Of the 470 patients included, 312 (66.4%) began therapy optimization during hospitalization, whereas 158 (33.6%) started therapy in the outpatient setting following discharge. All treatments were initiated and optimized according to guideline recommendations to ensure consistency across care settings.

### 2.3 Sample size determination and sampling technique

The study was designed to assess the time to first hospitalization among patients with cardiovascular disease using survival analysis. The sample size was calculated based on the Schoenfeld formula for Cox proportional hazards regression, assuming a cumulative event rate of 35% over a 12-month follow-up period [3,4,12]. To account for potential loss to follow-up (∼10%), the initial calculated sample size of 423 participants was increased to 470 participants.

Patients with a confirmed diagnosis of cardiovascular disease were recruited from the outpatient clinics and inpatient wards of three selected hospitals in Northwest Ethiopia. Individuals with terminal illnesses or those unable to provide reliable follow-up information were excluded to ensure data quality. The total sample was proportionally allocated to each hospital based on their patient load using the following formula:

$$n_{i}=\frac{N_{i}}{N_{total}}\times N$$

where n_i_ is the sample size for hospital i, N_i_​ is the patient load of hospital i, N total is the total patient load, and n is the total sample size. Based on this calculation, Felege Hiwot Hospital contributed 230 participants, Debre Markos Hospital 153, and Debre Tabor Hospital 87, achieving representative sampling across the three sites.

### 2.4 Study variables

The dependent variable was the time to the first cardiovascular hospitalization. Independent variables were categorized into four domains. Sociodemographic factors included age, sex, residence (urban or rural), education level, marital status, employment status, income level, and health insurance coverage. Lifestyle factors comprised smoking status (never, former, or current) and physical activity level (active, moderate, or sedentary). Clinical factors included the type of cardiovascular diagnosis (heart failure, hypertensive heart disease, ischemic heart disease, arrhythmias, cardiomyopathies, stroke/TIA, or other conditions), comorbidities such as hypertension, diabetes mellitus, dyslipidemia, obesity, chronic kidney disease, COPD, and family history of cardiovascular disease, as well as the Charlson Comorbidity Index (categorized as <3 vs. ≥3). Baseline clinical status was also considered, including left ventricular ejection fraction (<40% vs. ≥40%), presence of symptoms such as dyspnea, edema, chest pain, and prior ICU/CCU admission. Treatment-related factors included guideline-directed medical therapy at discharge (full vs. partial/none), medication adherence (good vs. poor, based on chart documentation and patient report), and the presence of a structured post-discharge follow-up plan. Guideline-based treatment status**,** categorized as optimal or suboptimal according to established clinical guidelines**,** was included as an independent variable.

### 2.5 Data collection instruments and procedures

Baseline information following treatment optimization was obtained from patient medical records. Sociodemographic and lifestyle characteristics were abstracted from chart documentation. Clinical information, including diagnoses, comorbidities, and relevant investigations, was extracted from inpatient and outpatient records. Medication prescriptions and adherence were recorded from discharge summaries and follow-up notes, relying on chart documentation and patient self-reports, as pharmacy refill data were unavailable. Data abstraction was conducted using a structured checklist adapted from previous literature. To ensure data quality, the tool was pretested on 5% of records, data collectors received standardized training, and supervisors performed daily cross-checks. All data were double-entered into EpiData version 4.6, and consistency checks were applied before analysis.

### 2.6 Follow-up and outcome measurement

Participants were followed for 12 months after treatment optimization, with the primary focus on the time to first cardiovascular hospitalization.

• Primary Outcome: Time to first cardiovascular hospitalization, defined as any hospital admission due to worsening heart failure, acute coronary syndrome, arrhythmia, stroke, or other physician-documented cardiovascular events, irrespective of prior hospitalization status.
• Secondary Outcomes:
  o In-hospital mortality
  o Intensive Care Unit (ICU) or Coronary Care Unit (CCU) admission

Cardiovascular hospitalizations were identified through patient medical records and hospital registries. Each hospitalization event was independently reviewed and adjudicated by two cardiologists to confirm that the admission was primarily due to a cardiovascular cause, with any discrepancies resolved by consensus discussion. Systematic follow-up, including review of records and patient contacts, ensured accurate estimation of hospitalization incidence and timing, thereby identifying predictors of early cardiovascular hospitalization.

### 2.7 Medication adherence assessment

Medication adherence was evaluated using chart review and patient self-report. Adherence was categorized as follows.

• Good adherence: Patients taking ≥80% of prescribed cardiovascular medications as documented or reported.
• Poor adherence: Patients taking <80% of prescribed medications, missing doses, or nonadherence to therapy schedules.

### 2.8 Quality assurance and missing data management

Data quality was ensured through standardized training of data collectors, pretesting of instruments, and daily cross-checks by field supervisors. Double data entry using EpiData version 4.6 minimized entry errors. Missing data were maintained below 5% for all variables, examined for randomness, and handled using complete-case analysis (see Supplementary Table S1). All data were anonymized to protect confidentiality. The proportional hazards assumption for the Cox regression model was verified using Schoenfeld residuals and met (p > 0.05).

### 2.9 Data entry, management, and statistical analysis

Data were entered using EpiData version 4.6 and analyzed in STATA version 17. Continuous variables were assessed for normality using the Shapiro–Wilk test, and categorical variables were summarized as frequencies and percentages.

Time-to-event analysis: Kaplan–Meier survival curves were constructed to estimate the probability of remaining free from cardiovascular hospitalization. Kaplan–Meier curves were generated to illustrate the time to the first cardiovascular hospitalization stratified by GDMT status, with comparisons made using the log-rank test.

Multivariate analysis was performed using the Cox proportional hazards model, with results expressed as hazard ratios (HRs) and 95% confidence intervals (CIs). Care setting at treatment optimization (inpatient vs. outpatient) was included as a covariate to account for baseline differences in clinical severity. A sensitivity analysis stratified by prior hospitalization status was also conducted.

Missing data were minimal (<5% for all variables) and assumed to be missing at random; a complete-case analysis was performed. Multicollinearity among covariates was assessed using variance inflation factors (VIF), with all VIF values < 5, indicating no significant multicollinearity.

To account for the competing risk of death on cardiovascular hospitalization and readmission, we applied the Fine–Gray subdistribution hazard model, estimating cumulative incidence while treating death as a competing event. Results are presented as subdistribution hazard ratios (sHRs) with 95% CIs, and cumulative incidence functions were plotted to illustrate differences across key covariates.

### 2.10 Ethics approval and consent to participate

The Institutional Review Board of Debre Tabor University approved the study (Ref No: 298/2023). Participants were informed about the study objectives and provided written informed consent before enrollment, with the option to withdraw at any time without consequence. The study ensured confidentiality by omitting personal identifiers and assigning unique codes to each participant. All data were handled in accordance with the ethical guidelines and the Declaration of Helsinki.

### 2.11 Operational definitions

• **Time to First Cardiovascular Hospitalization:** Number of days from treatment optimization to the first hospital admission for cardiovascular causes within 12 months. Participants who did not experience hospitalization were censored at the end of follow-up or at death.
• **Treatment Optimization:** Initiation, titration, or adjustment of cardiovascular therapy including pharmacologic and guideline-recommended non-pharmacologic interventions according to the 2022 AHA/ACC/HFSA Heart Failure Guidelines [10] and Cardiovascular guidelines recommend evidence-based medications, risk factor control, symptom management, arrhythmia treatment, device therapy when needed, and structured follow-up with lifestyle interventions. Dose titration was based on achieving either the recommended target dose or the maximally tolerated dose, considering patient tolerance, vital signs, renal function, and laboratory parameters.
• **Guideline-based treatment optimization** was assessed using patient medical records and classified as optimal or suboptimal based on the prescription of diagnosis-appropriate, evidence-based pharmacologic therapy in accordance with international and national cardiovascular guidelines. Treatment optimization was evaluated relative to the specific cardiovascular diagnosis and clinical indication and was used as an indicator of guideline-concordant care.
• **Cardiovascular Hospitalization:** Any hospital admission due to heart failure, acute coronary syndrome, arrhythmias, stroke, or other clinically significant cardiovascular events, including procedures such as percutaneous coronary intervention (PCI) or coronary artery bypass grafting (CABG).
• Charlson Comorbidity Index: A validated scoring system quantifying the burden of comorbid diseases to predict mortality risk.
• Current smoker: A patient actively using tobacco products at the time of study enrollment.
• Rehospitalization: Any unplanned hospitalization within 12 months after discharge for cardiovascular causes.
• Guideline-Directed Medical Therapy (GDMT) Assessment: GDMT components included ACE inhibitors/ARBs/ARNIs, beta-blockers, mineralocorticoid receptor antagonists (MRAs), and SGLT2 inhibitors. For hospitalized patients, GDMT status was assessed at discharge; for outpatients, it was determined at the most recent clinic visit. Therapy status was categorized as
  o Full: All indicated therapies prescribed at target or maximally tolerated doses.
  o Partial/None: Any indicated therapy missing or prescribed below the recommended doses.

*Note:* Low GDMT utilization in this cohort reflects the limited availability of certain therapies in Ethiopia's essential medicine list during the study period and the inclusion of patients with diverse cardiovascular conditions, not all of whom required all GDMT components.

## 3 Results

### 3.1 Study population

**Fig. 1:**
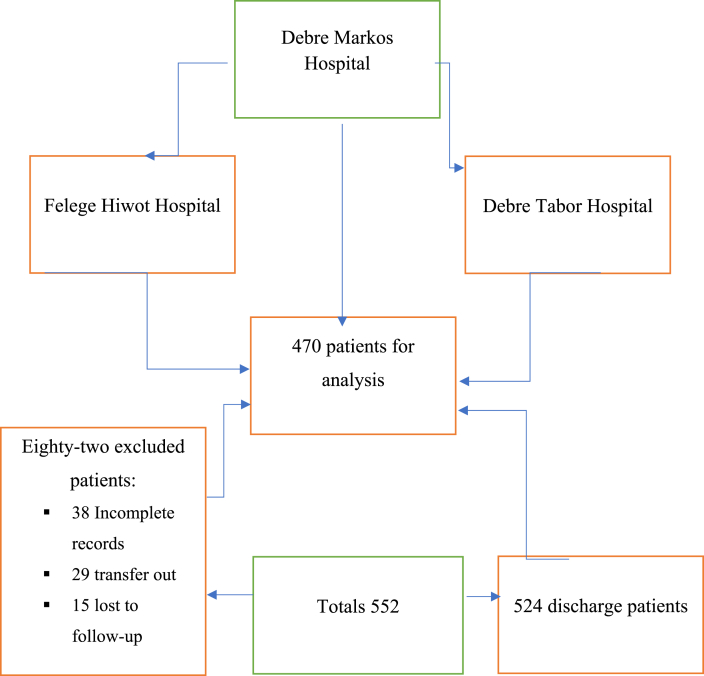
Flow diagram of the study participants from assessment to inclusion at the three selected hospitals in Northwest Ethiopia.

A study involving 552 patients with cardiovascular disease in Northwest Ethiopia, excluding 82, 38 with incomplete records, 29 transferred out, and 15 lost to follow-up, included 470 patients in the final analysis (Fig. 1).

### 3.2 Patient demographic characteristics

The study included 470 patients with cardiovascular disease. Most participants were younger than 65 years and were predominantly male. The majority were covered by health insurance and were distributed across low-, middle-, and high-income categories. Most patients were married, had primary-level education, and reported moderate to active lifestyles. The majority had no family history of heart failure and were non-smokers. Treatment optimization occurred during hospitalization in 312 patients (66.4%), while 158 patients (33.6%) underwent optimization in the outpatient setting (Table 1).

**Table 1:** Sociodemographic and behavioral characteristics of patients with cardiovascular disease (N = 470) in Northwest Ethiopia, 2023.

Variable	Category	Frequency n (%)
Age (years) mean		59.8 ± 13.6
Age	65	356 (75.4)
	≥65	114 (24.6)
Sex	Male	255 (54.3)
	Female	215 (45.7)
Residence	Rural area	290 (61.7)
	Urban area	180 (38.3)
Health insurance	Insured	276 (58.7)
	Uninsured	194 (41.3)
Income level	Low	198 (42.1)
	Middle	186 (39.6)
	high	86 (18.3)
Employment status	Employed	152 (32.3)
	Unemployed	128 (27.2)
	Retired	84 (17.9)
	Student	39 (8.3)
	Homemaker/Other	67 (14.3)
Marital status	Married	318 (67.7)
	Divorced	28 (6.0)
	Single	64 (13.6)
	Widowed	48 (10.2)
	Other	12 (2.6)
Education status	No formal education	141 (30.0)
	Primary	154 (32.8)
	Secondary	108 (23.0)
	Tertiary/University	67 (14.3)
Family history of heart failure	Yes	112 (23.8)
	No	358 (76.2)
Physical activity level	Active	102 (21.7)
	Moderate	168 (35.7)
	Sedentary	200 (42.6)
Cigarette Smoking	Never smoked	335 (71.3)
	Former smoker	74 (15.7)
	Current smoker	61 (13.0)
Total		470 (100.0)

Footnotes.

1. HF = heart failure.

2. Physical activity levels were self-reported and categorized as active, moderate, or sedentary according to the operational definitions in the Methods section.

3. Income levels were classified based on patient-reported monthly household income (low, middle, and high).

4. NYHA functional class and baseline blood pressure and heart rate are included in Table 1. BNP/NT-proBNP measurements were not routinely performed in the participating centers and are therefore unavailable for this cohort, which may limit the full assessment of baseline cardiac risk.

### 3.3 Clinical characteristics

The study evaluated 470 patients, revealing the most common cardiovascular diagnoses as heart failure (57.0%), hypertensive heart disease (27.2%), and ischemic heart disease (23.8%), with leading risk factors and comorbidities including hypertension, diabetes, smoking, dyslipidemia, and obesity (Table 2).

**Table 2:** Clinical diagnoses, comorbidities, and symptoms of patients with cardiovascular disease (N = 470) in Northwest Ethiopia, 2023.

Category	Variable/Diagnosis	Frequency n (%)
Cardiovascular Diagnoses	Ischemic heart disease (stable angina, ACS, and MI)	112 (23.8)
	Hypertensive heart disease (LVH, hypertensive HF)	128 (27.2)
	Heart failure (HFrEF + HFpEF)	268 (57.0)
	Cardiomyopathies (dilated, hypertrophic, restrictive)	24 (5.1)
	Arrhythmias (AF, VT, and heart block)	64 (13.6)
	Congenital heart disease	8 (1.7)
	Peripheral artery disease	19 (4.0)
	Cerebrovascular disease (stroke, TIA)	52 (11.1)
	Pulmonary hypertension	12 (2.6)
	Aortic disease (aneurysm, dissection)	6 (1.3)
	Pericardial diseases (pericarditis and effusion)	10 (2.1)
Cardiovascular risk factors/comorbidities	Hypertension	274 (58.3)
	Diabetes mellitus	146 (31.1)
	Dyslipidemia	98 (20.9)
	Smoking history	135 (28.7)
	Obesity (BMI ≥30 kg/m^2^)	72 (15.3)
	Family history of CVD	112 (23.8)
	Chronic kidney disease	58 (12.3)
	COPD	39 (8.3)
Clinical Symptoms/Signs	Chest pain/angina	128 (27.2)
	Dyspnea (exertional or at rest)	268 (57.0)
	Fatigue	212 (45.1)
	Palpitations	92 (19.6)
	Syncope/dizziness	48 (10.2)
	Ankle edema	152 (32.3)
	Nocturnal cough	36 (7.7)
	Hepatomegaly	29 (6.2)
	Tachycardia	64 (13.6)

Footnotes.

1. ACS = acute coronary syndrome; MI = myocardial infarction; LVH = left ventricular hypertrophy; HFrEF = heart failure with reduced ejection fraction; HFpEF = heart failure with preserved ejection fraction; AF = atrial fibrillation; VT = ventricular tachycardia; TIA = transient ischemic attack; COPD = chronic obstructive pulmonary disease.

2. Patients may have multiple diagnoses, comorbidities, and symptoms; percentages do not sum to 100%.

3. Obesity is defined as BMI ≥30 kg/m^2^.

### 3.4 Hospitalization and readmission outcomes

A study of 470 patients found that the mean hospital stay was 7.8 days, with 78 patients requiring ICU/CCU care. Heart failure decompensation was the leading cause of hospitalization, followed by ischemic events, arrhythmia, stroke/TIA, and other cardiovascular causes. Most hospitalizations were emergency admissions, and 34.9% experienced readmission. Full guideline-directed medical therapy was received by 48.1% of the patients (Table 3).

**Table 3:** Hospitalization outcomes and readmission characteristics of patients with cardiovascular disease (n = 470).

Variable	Category/Metric	Frequency n (%)/Median (IQR)
Length of stay (days)	Mean ± SD	7.8 ± 4.0
ICU/CCU admission	Yes	78 (16.6)
Discharge therapy	Full GDMT	226 (48.1)
	Partial/No GDMT	244 (51.9)
Follow-up plan at discharge	Scheduled	358 (76.2)
	Not scheduled	112 (23.8)
First hospitalization after discharge	(n = 218)	
Time to the first hospitalization (days)	Median (IQR)	100 (55–160)
Type of hospitalization	Emergency	153 (70.2)
	Elective/planned	65 (29.8)
Primary cause	Heart failure decompensation	131 (60.1)
	Ischemic events (MI/angina)	44 (20.2)
	Arrhythmia	17 (7.8)
	Stroke/TIA	13 (6.0)
	Other cardiovascular causes	13 (6.0)
Length of stay (days)	Median (IQR)	7 (4–11)
Hospitalization within 1 year	Yes	218 (46.4)
	No	252 (53.6)
Readmission within 1 year	Yes	164 (34.9)
	No	306 (65.1)
Number of readmissions (n = 164)	1 time	102 (62.2)
	≥2 times	62 (37.8)
Time to the first readmission (days)	Median (IQR)	124 (72–198)
Cause of first readmission (n = 164)	Heart failure decompensation	96 (58.5)
	Ischemic events (MI/angina)	28 (17.1)
	Arrhythmia	18 (11.0)
	Stroke/TIA	12 (7.3)
	Other CV causes	10 (6.1)
Length of stay during readmission (days)	Median (IQR)	7 (5–11)
In-hospital mortality during readmission (n = 164)	Yes	21 (12.8)
	No	143 (87.2)

Footnotes.

1. ICU = intensive care unit; CCU = coronary care unit; GDMT = guideline-directed medical therapy; MI = myocardial infarction; TIA = transient ischemic attack.

2. Percentages for first hospitalization were calculated among patients hospitalized within 1 year (n = 218); percentages for readmission were calculated among patients readmitted within 1 year (n = 164).

3. Patients may have multiple causes or conditions; percentages do not sum to 100%.

4. Emergency hospitalization refers to unplanned admissions; elective/planned refers to scheduled admissions.

### 3.5 Medication optimization and prescription patterns

The most commonly prescribed medication class among the 470 patients with cardiovascular disease was diuretics (44.9%), primarily for symptom relief and volume management. Other commonly used medications included antiplatelets, lipid-lowering agents, and anticoagulants. Comorbid conditions such as antibiotics, bronchodilators, ferrous sulfate, and hypoglycemic agents were also frequently prescribed (Table 4).

**Table 4:** Medication prescription patterns among cardiovascular disease patients (N = 470), Northwest Ethiopia, 2023.

Drug class	Category	Frequency	Percentage (%)	Comment/Relevance
ACEI/ARB	Prescribed	175	37.2	Core GDMT for HFrEF and hypertension
Beta-blocker	Prescribed	121	25.7	GDMT for HFrEF, arrhythmia, and IHD
MRA (spironolactone, eplerenone)	Prescribed	147	31.3	GDMT for HFrEF
SGLT2 inhibitors	Prescribed	31	6.6	Emerging therapy for HFrEF/diabetes
ARNI	Prescribed	4	0.9	Advanced GDMT (sacubitril/valsartan)
Diuretics	Prescribed	211	44.9	Symptomatic relief and volume management
Anticoagulants	Prescribed	45	9.6	Atrial fibrillation and thromboembolism
Antiplatelets	Prescribed	98	20.9	IHD and post-ACS management
Lipid-lowering agents	Prescribed	86	18.3	Statins for IHD/secondary prevention
Digitalis/inotropes	Prescribed	19	4.0	Symptomatic HFrEF support
Antibiotics	Prescribed	84	17.9	Comorbidity-driven (e.g., infection)
Calcium channel blockers	Prescribed	38	8.1	Hypertension/angina
Ferrous sulfate	Prescribed	29	6.2	Anemia management
Hypoglycemic agents	Prescribed	14	3.0	Diabetes comorbidity
Bronchodilators/inhaled corticosteroids	Prescribed	22	4.7	COPD/asthma comorbidity
Others	Prescribed	52	11.1	Other adjunct therapies

Footnotes.

1. Abbreviations: ACEI = angiotensin-converting enzyme inhibitor; ARB = angiotensin receptor blocker; MRA = mineralocorticoid receptor antagonist; SGLT2 = sodium-glucose cotransporter 2 inhibitor; ARNI = angiotensin receptor-neprilysin inhibitor; GDMT = guideline-directed medical therapy; HFrEF = heart failure with reduced ejection fraction; IHD = ischemic heart disease; ACS = acute coronary syndrome; COPD = chronic obstructive pulmonary disease.

2. Multiple prescriptions: Patients may have received more than one medication; percentages do not sum to 100%.

3. Comorbidity medications: Antibiotics, hypoglycemic agents, bronchodilators, ferrous sulfate, and other adjunct therapies were prescribed for non-cardiovascular comorbidities.

4. Emerging/advanced therapies: SGLT2 inhibitors and ARNI have recently been recommended as GDMT for HFrEF and are often underutilized in resource-limited settings.

### 3.6 Predictors of the first cardiovascular hospitalization

The study found that several factors, including partial or no guideline-directed medical therapy at discharge, a high Charlson Comorbidity Index, and lack of a scheduled follow-up plan, were independently associated with a shorter time to first cardiovascular hospitalization within 1 year. The other significant predictors included heart failure with reduced ejection fraction, chronic kidney disease, current smoking, age ≥65 years, and low physical activity (Table 5).

**Table 5:** Multivariate Cox regression: Predictors of time to first cardiovascular hospitalization within 1 year (N = 470).

Predictor	Category	Adjusted HR	95% CI	p-value
Age	≥65 vs < 65	1.36	1.05–1.76	0.020
Sex	Male vs. Female	1.09	0.85–1.40	0.490
Residence	Rural vs. Urban	1.22	0.95–1.56	0.110
Education	No formal vs ≥ Primary	1.18	0.88–1.58	0.280
Income level	Low vs. Middle/High	1.25	0.94–1.66	0.120
Physical activity	Low vs. Moderate/High	1.35	1.03–1.77	0.029
Smoking status	Current vs. Never/Former	1.40	1.01–1.95	0.043
Hypertension	Yes vs. No	1.18	0.89–1.56	0.250
Diabetes mellitus	Yes vs. No	1.28	0.97–1.69	0.080
Chronic kidney disease	Yes vs. No	1.41	1.02–1.95	0.036
Previous stroke/TIA	Yes vs. No	1.31	0.90–1.91	0.160
Charlson comorbidity index	≥3 vs < 3	1.62	1.25–2.10	<0.001
Heart failure	LVEF <40% vs ≥ 40%	1.46	1.10–1.94	0.008
ACS/ischemic heart disease	Yes vs. No	1.12	0.82–1.53	0.460
Arrhythmia	Yes vs. No	1.20	0.85–1.70	0.290
Valvular disease	Yes vs. No	1.08	0.65–1.80	0.760
ICU/CCU admission	Yes vs. No	1.28	0.95–1.73	0.100
GDMT at discharge	Partial/None vs. Full	1.71	1.30–2.25	<0.001
Follow-up plan	None vs. Scheduled	1.60	1.18–2.16	0.003

Footnotes.

1. HR = hazard ratio; CI = confidence interval; HFrEF = heart failure with reduced ejection fraction; LVEF = left ventricular ejection fraction; GDMT = guideline-directed medical therapy; CCI = Charlson Comorbidity Index; ACS = acute coronary syndrome; TIA = transient ischemic attack; ICU/CCU = intensive/coronary care unit.

2. HR > 1 indicates a higher hazard (shorter time) to the first cardiovascular hospitalization.

3. Comparison groups are indicated in the Category column; e.g., partial/none vs. full GDMT, none vs. scheduled follow-up plan.

4. Multivariate Cox regression adjusted for all the variables listed.

5. Variables with p < 0.05 are considered statistically significant predictors.

### 3.7 Kaplan–Meier analysis of guideline adherence and time to first cardiovascular hospitalization

The Kaplan–Meier survival curves illustrate the time to the first cardiovascular hospitalization stratified by adherence to guideline-based therapy at discharge. Patients who adhered fully to guideline-based therapy demonstrated a higher probability of remaining free from hospitalization over the follow-up period compared to those with partial or no adherence. The separation of the survival curves indicates a protective effect of adherence to guideline-based therapy. The shaded areas represent 95% confidence intervals, which show some overlap at later follow-up times, indicating reduced precision due to fewer patients at risk. Overall, the plot visually supports the finding that non-adherence to guideline-based therapy is associated with earlier cardiovascular hospitalization.

### 3.8 Competing risk analysis

The study involved 470 patients with cardiovascular conditions. Key findings indicate that individuals aged ≥65 years had a higher risk of initial cardiovascular hospitalization (sHR 1.32, 95% CI 1.03–1.70, p = 0.028). Lifestyle factors, such as low physical activity (sHR 1.33, 95% CI 1.01–1.75, p = 0.041) and current smoking (sHR 1.37, 95% CI 0.99–1.90, p = 0.048), were positively associated with hospitalization risk. Chronic kidney disease (sHR 1.38, 95% CI 1.00–1.92, p = 0.048) and a Charlson Comorbidity Index ≥3 (sHR 1.58, 95% CI 1.21–2.06, p < 0.001) were significant predictors, whereas other comorbidities like hypertension and diabetes were not. Clinically, heart failure with reduced ejection fraction (LVEF <40%) led to increased hospitalization risk (sHR 1.43, 95% CI 1.07–1.91, p = 0.015). Furthermore, inadequate guideline-directed medical therapy at discharge increased hospitalization risk by 1.68 times (95% CI 1.28–2.21, p < 0.001), and lack of a follow-up plan increased risk by 1.57 times (95% CI 1.15–2.14, p = 0.004)Table 6.

**Table 6:** Predictors of first cardiovascular hospitalization accounting for death as a competing risk.

Predictor	Category	Sub-distribution HR	95% CI	p-value
Demographics				
Age	≥65 vs < 65	1.32	1.03–1.70	0.028
Sex	Male vs. Female	1.07	0.83–1.38	0.600
Residence	Rural vs. Urban	1.18	0.91–1.53	0.210
Education	No formal vs ≥ Primary	1.16	0.87–1.55	0.310
Income level	Low vs. Middle/High	1.23	0.92–1.64	0.160
Physical activity	Low vs. Moderate/High	1.33	1.01–1.75	0.041
Smoking status	Current vs. Never/Former	1.37	0.99–1.90	0.048
Comorbidities				
Hypertension	Yes vs. No	1.15	0.87–1.53	0.320
Diabetes mellitus	Yes vs. No	1.26	0.95–1.67	0.100
Chronic kidney disease	Yes vs. No	1.38	1.00–1.92	0.048
Previous stroke/TIA	Yes vs. No	1.28	0.87–1.88	0.210
Charlson comorbidity index	≥3 vs < 3	1.58	1.21–2.06	<0.001
Clinical Factors				
Heart failure	LVEF <40% vs ≥ 40%	1.43	1.07–1.91	0.015
ACS/ischemic heart disease	Yes vs. No	1.10	0.80–1.50	0.560
Arrhythmia	Yes vs. No	1.18	0.83–1.68	0.340
Valvular disease	Yes vs. No	1.05	0.63–1.75	0.850
ICU/CCU admission	Yes vs. No	1.24	0.91–1.68	0.170
Treatment and follow-up				
GDMT at discharge	Partial/None vs. Full	1.68	1.28–2.21	<0.001
Follow-up plan	None vs. Scheduled	1.57	1.15–2.14	0.004

Footnotes: 1. sHR = sub-distribution hazard ratio; CI = confidence interval. 2. Analyses were performed using Fine–Gray subdistribution hazard models, treating death as a competing event. 3. Statistically significant predictors (p < 0.05) are highlighted in bold. 4. HFrEF = heart failure with reduced ejection fraction (LVEF <40%). 5. Charlson Comorbidity Index (CCI) ≥3 indicates a high comorbidity burden.

## 4 Discussion

In a multicenter retrospective cohort study in Northwest Ethiopia, 46.4% of patients with cardiovascular disease experienced hospitalization within a year post-treatment optimization, with a median admission time of 100 days. The study identified a vulnerable period within the first 3–4 months after optimization, highlighting risks associated with partial medical therapy, high comorbidity, reduced left ventricular ejection fraction, chronic kidney disease, smoking, low physical activity, older age, and lack of follow-up plans. These findings underscore the need for structured monitoring and adherence to evidence-based therapies to reduce early rehospitalization in resource-limited settings.

### 4.1 Prevalence and systemic factors

The observed prevalence of early cardiovascular hospitalization in the current cohort, at 46.4% within one-year post-treatment optimization, indicates the substantial burden of recurrent cardiovascular events in this population. This rate is notably higher than some reports from high-income countries, where re-hospitalization rates for cardiovascular diseases typically range between 25% and 40% within one year [2,10]. Conversely, similar or even higher rates have been documented in other low- and middle-income countries (LMICs), owing to the influence of resources constraint and healthcare disparities [3,13]. The elevated prevalence in this setting may be attributed to factors such as limited access to comprehensive outpatient management, suboptimal medication adherence, and delayed diagnosis or treatment of comorbidities. Additionally, infrastructure challenges, such as the shortage of trained healthcare providers, inadequate patient education, and lack of formal follow-up systems, likely contribute to this high rehospitalization burden. These findings emphasize the urgent need for health system strengthening and targeted interventions to reduce early cardiovascular hospital admissions in Ethiopia and comparable settings.

### 4.2 Guideline-based therapy

The current study reported that the key finding was that partial or no adherence to GDMT at discharge increased hospitalization risk by 71%. This is consistent with extensive evidence from recent clinical trials and registries demonstrating that optimal GDMT significantly reduces cardiovascular mortality and morbidity, particularly in patients with heart failure [4,6,14]. The CHAMP-HF registry showed that patients receiving suboptimal GDMT had higher rates of adverse cardiovascular events. However, the current study revealed notably low GDMT adherence, with only 25.7% receiving beta blockers and fewer than 10% treated with newer agents such as SGLT2 inhibitors and ARNIs. This contrasts sharply with higher incomes, where GDMT uptake is substantially higher [7,15]. These disparities likely reflect systemic barriers in LMICs, including drug unavailability, high medication costs, and limited provider familiarity or confidence with newer therapies [8,16]. Structural healthcare challenges, such as inconsistent drug supply chains and lack of guideline dissemination, may also contribute. Addressing these barriers through policy reforms, subsidized drug programs, and continuing medical education could significantly reduce hospitalization rates.

In this study, the utilization of GDMT among patients with HFrEF was significantly low, with only 0.9% receiving ARNI, 6.6% receiving SGLT2 inhibitors, and 25.7% receiving beta-blockers. Contributing factors include the limited availability of certain medications in Ethiopia's essential medicines list and the inclusion of patients not strictly diagnosed with HFrEF. GDMT was defined as triple therapy (renin–angiotensin–aldosterone system inhibitor, beta-blocker, and mineralocorticoid receptor antagonist), with quadruple therapy including an SGLT2 inhibitor. This underutilization highlights the need for better access to cardiovascular medications and adherence to evidence-based treatment protocols to enhance patient outcomes.

### 4.3 Kaplan–Meier analysis and competing risk findings

**Fig. 2:**
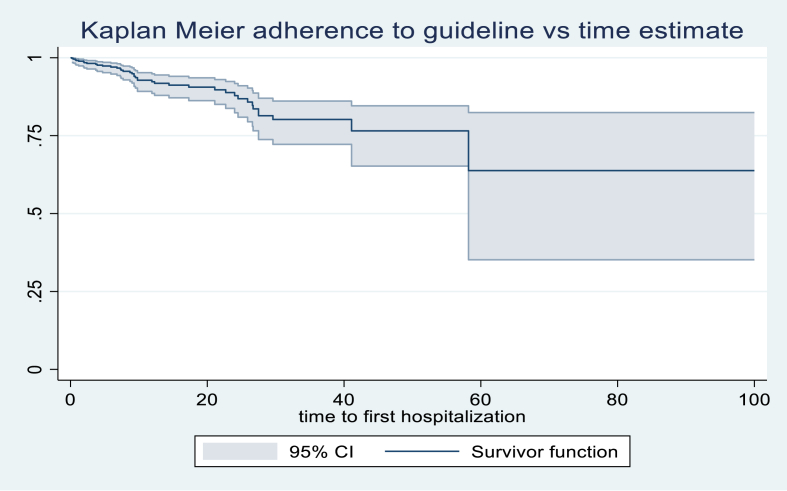
Kaplan–Meier survival curves for the time to the first cardiovascular hospitalization stratified by guidelines at discharge. Footnotes: *1*. *Full guideline-based therapy includes all indicated cardiovascular treatments prescribed at target or maximally tolerated doses.* Guideline-based therapy is individualized based on patient comorbidities, renal function, blood pressure, and tolerability. In patients with heart failure and post-MI, adherence to guideline therapy has been consistently shown to reduce hospitalization, cardiovascular events, and mortality). 2. Partial/no guideline-based therapy indicates missing one or more indicated therapies or subtherapeutic dosing. 3. Censoring occurred for patients who did not experience hospitalization or who died during follow-up.

Kaplan–Meier survival curves (Fig. 2) and competing risk analysis (Table 6) further illustrate the impact of GDMT adherence and other patient-level factors on time to first cardiovascular hospitalization. Patients with full adherence to guideline-based therapy had a higher probability of remaining free from hospitalization throughout the follow-up period, whereas those with partial or no adherence experienced earlier events. The Fine-Gray competing risk analysis confirmed that age ≥65 years, low physical activity, current smoking, chronic kidney disease, Charlson Comorbidity Index ≥3, HFrEF, partial/no GDMT, and absence of a follow-up plan were all significant independent predictors of earlier hospitalization. These findings reinforce the importance of both patient-level factors and system-level interventions, such as structured follow-up, in reducing early rehospitalizations in resource-limited settings.

### 4.4 Comorbidity and clinical risk factors

Patients with a Charlson Comorbidity Index (CCI) ≥ 3 had a 62% higher risk of hospitalization. The CCI remains a validated predictor of mortality and re-hospitalization in cardiovascular cohorts globally [9,17]. Multimorbidity complicates clinical management, increasing the risk of adverse drug interactions, polypharmacy, and disease exacerbations. Our findings align with recent studies showing that patients with multiple chronic conditions have worse outcomes and higher healthcare use [18]. In high-income settings, integrated multidisciplinary care models improve the outcomes of multimorbid patients [5]. However, such coordinated care is often lacking in LMICs due to workforce shortages, fragmented systems, and limited access to specialty care, which likely contributed to the increased hospitalizations in our cohort. Smoking demonstrated borderline statistical significance**,** implied a possible contribution to rehospitalization risk; however, the confidence interval approached unity, and the finding should therefore be interpreted cautiously. Larger prospective studies are warranted to clarify its independent effect within competing risk frameworks.

### 4.5 Post-discharge follow-up

This study recorded that the absence of a schedule post-discharge follow-up plan was associated with a 60% increased hospitalization risk, underscoring the critical role of structured transitional care. Recent random controlled trials and meta-analyses have confirmed that coordinated post-discharge interventions reduce re-hospitalization and mortality in heart failure and other cardiovascular populations [19,20]. In many LMICs, logistical challenges, such as transport costs and geographic barriers to outpatient clinic capacity, hinder effective follow-up [21]. In this study, the findings likely reflect these systemic obstacles. Innovative solutions such as telemedicine, community health worker involvement, and mobile health interventions may improve follow-up adherence and reduce rehospitalizations in resource-constrained settings.

### 4.6 Cardiac function and organ dysfunction

Based on the current study report, reduced LVEF was a significant predictor of hospitalization, consistent with the extensive literature link to systolic dysfunction to morbidity and mortality [21,22]. Landmark trials and updated guidelines have reinforced that patients with LVEF<40% are at a higher risk of hospitalization and death [23,24]. The current findings also reinforce the importance of targeted management of systolic heart failure. The low use of guideline-recommended agents such as ARNIs and SGLTI2 inhibitors in this population may have contributed to the elevated hospitalization risk, as these medications have been shown to substantially reduce heart failure with other comorbidities hospitalization [25]. Limited access to or affordability of these medications in LMICs remains a significant barrier to optimal care.

Chronic kidney disease (CKD) independently increased hospitalization risk, consistent with multiple recent studies demonstrating that kidney dysfunction worsens cardiovascular prognosis through mechanisms including volume overload, electrolyte disturbance, and accelerated vascular disease [26,27]. In resource-limited settings, such as Ethiopia, limited access to nephrology services and renal replacement therapies may worsen the outcomes for patients with CKD, contributing to increased cardiovascular hospitalizations. Early identification and integrated management of CKD in cardiovascular patients are essential to mitigate the risks.

### 4.7 Lifestyle and demographic factors

This study reported that current smoking was associated with increased hospitalization risk, consistent with global data linking tobacco use to accelerate cardiovascular disease progression and worse outcomes [28,29]. Smoking cessation remains a cornerstone of cardiovascular disease prevention, yet programs are often underdeveloped in LMICs [30]. Similarly, low physical activity independently predicted hospitalization, reflecting the well-established protective effects of regular exercise on cardiovascular health [31,32]. Socioeconomic, cultural, and environmental barriers likely contributed to low physical activity levels. These findings emphasize the need to incorporate culturally appropriate lifestyle interventions, including smoking cessation support and physical activity promotion, into cardiovascular care.

Older age (≥65 years) was associated with increased hospitalization risk, consistent with numerous studies reporting age as a non-modifiable risk factor for adverse cardiovascular outcomes [33]. Older adults often present with greater comorbidity burden, frailty, and polypharmacy, which complicate management and increase the risk of hospitalization. Although age cannot be changed, geriatric-focused care models and comprehensive risk assessments may help tailor interventions to reduce hospitalizations in this vulnerable group.

### 4.8 System-level and contextual factors

These findings reveal parallels with global predictors of cardiovascular hospitalizations while emphasizing significant treatment gaps and systemic challenges present in low- and middle-income countries (LMICs). Notably, the low uptake of guideline-directed medical therapy (GDMT) and advanced therapies contrasts sharply with higher-resource environments. Factors such as economic constraints, medication shortages, inadequate healthcare infrastructure, and insufficient provider training contribute to this issue. The prevalence of multimorbidity and lack of structured follow-up further indicate fragmented care and workforce shortages. Additionally, social determinants like poverty and geographic barriers likely heighten risks, although these were not explicitly evaluated in this study. Future research should address these elements to develop suitable interventions. The limited adoption of advanced therapies, such as SGLT2 inhibitors and ARNIs, reflects systemic barriers, including high costs and lack of provider familiarity, notably in resource-limited settings like Ethiopia.

### 4.9 Cohort heterogeneity

Inclusion of both inpatient and outpatient populations enhances the external validity of the findings by reflecting real-world cardiovascular care in a low-resource setting. Although this approach introduces clinical heterogeneity, potential bias was mitigated through clear outcome definition and adjustment for care setting in multivariate analyses.

### 4.10 Strengths and limitations

This study highlights strengths such as its multicenter design and large sample size (n = 470), which enhances the representativeness of cardiovascular patients in Northwest Ethiopia. It addresses knowledge gaps in low-resource settings by providing context-specific evidence on hospitalization predictors. The study used thorough data collection and adjustment for covariates to improve causal interpretations through competing risk analyses. However, limitations include potential bias from retrospective design and reliance on patient-reported medication adherence, lack of independent verification for outcome adjudication, and an operational definition of treatment optimization based on local guidelines. Additionally, inconsistencies in reporting prognostic variables, a confined study population limited to tertiary hospital attendees, and a complete-case analysis may affect the results' generalizability and statistical power. The inclusion of both first-time hospitalized and outpatient-managed patients may also blur the lines in distinguishing rehospitalization predictors from first hospitalization factors.

## 5 Conclusion

First cardiovascular hospitalizations are common following treatment optimization in northwest Ethiopia, with a median time to admit of 100 days. Risk factors include partial or no adherence to guideline-based therapy, high comorbidity burden (CCI ≥3), reduced LVEF, chronic kidney disease, absence of scheduled post-discharge follow-up, smoking, low physical activity, and older age. These findings highlight gaps in treatment implementation and follow-up, emphasizing the need for strengthened guideline-based management and structured care to prevent early hospitalizations in this setting.

## CRediT authorship contribution statement

**Getachew Yitayew Tarekegn:** Writing – review & editing, Writing – original draft, Visualization, Validation, Supervision, Software, Resources, Project administration, Methodology, Investigation, Funding acquisition, Formal analysis, Data curation, Conceptualization. **Abel Temeche Kassaw:** Writing – review & editing, Writing – original draft, Visualization, Resources, Project administration, Funding acquisition. **Tilaye Arega Moges:** Writing – review & editing, Writing – original draft, Formal analysis, Data curation. **Samuel Agegnehu Wondm:** Writing – review & editing, Writing – original draft, Data curation, Conceptualization. **Samuel Berihun Dagnew:** Writing – review & editing, Writing – original draft, Validation, Supervision. **Tigabu Eskeziya Zerihun:** Writing – review & editing, Writing – original draft, Supervision, Methodology. **Desalegn Addis Mussie:** Writing – review & editing, Writing – original draft, Resources, Project administration. **Fasil Bayafers Tamene:** Writing – review & editing, Writing – original draft, Project administration, Methodology. **Abaynesh Fentahun Bekalu:** Writing – review & editing, Writing – original draft, Validation. **Woretaw Sisay zewdu:** Writing – review & editing, Writing – original draft, Validation, Supervision.

## Clinical trial numbers

Not applicable.

## Funding agents

There were no funding agents for this research.

## Conflicts of interest

The authors declare that there were no conflicts of interest.

## Data Availability

Data will be available from the corresponding author up on reasonable request.

## Abbreviations and acronyms

ACS: Acute Coronary Syndrome
ACEI: angiotensin-converting enzyme inhibitor
AF: Atrial Fibrillation
ARB: angiotensin receptor blocker
ARNI: Angiotensin Receptor-Neprilysin Inhibitor
BMI: Body mass index
CCU: Coronary Care Unit
CI: confidence interval
CKD: Chronic Kidney Disease
COPD: Chronic Obstructive Pulmonary Disease
CVD: cardiovascular disease
CCI: Charlson Comorbidity Index
CV: Cardiovascular
ED: Emergency Department (if applicable)
GDMT: Guideline-Directed Medical Therapy
HF: heart failure
HFrEF: Heart failure with reduced EF
HFpEF: Heart failure with preserved ejection fraction
HR: Hazard ratio
ICU: Intensive Care Unit
IHD: Ischemic heart disease
IQR: Interquartile Range
LMICs: Low- and Middle-income countries
LVH: Left Ventricular Hypertrophy
LVEF: Left Ventricular Ejection Fraction
MI: Myocardial infarction
MRA: mineralocorticoid receptor antagonist
PCI: Percutaneous Coronary Intervention
PO Box: Post Office Box
SGLT2: Sodium-Glucose Cotransporter 2 Inhibitor
SD: standard deviation
TIA: Transient Ischemic Attack
USA: United States of America
