# Supplementary material for: Time to first cardiovascular hospitalization after guideline-based treatment optimization: A multicenter retrospective cohort study in northwest Ethiopia
Source: Int J Cardiol Cardiovasc Risk Prev. 2026 Feb 17;29:200607. doi: 10.1016/j.ijcrp.2026.200607 (PMC12938863; doi:10.1016/j.ijcrp.2026.200607)
Supplement: Multimedia component 1 [file mmc1.docx]

**Supplementary Table S1. Proportion of missing data by variable**

| **Variable** | **Missing n** | **Missing %** | **Handling Strategy** |
| --- | --- | --- | --- |
| Age | 0 | 0% | Complete-case |
| Sex | 0 | 0% | Complete-case |
| Baseline NYHA class | 5 | 1.1% | Complete-case |
| Systolic BP | 10 | 2.1% | Complete-case |
| Diastolic BP | 10 | 2.1% | Complete-case |
| Heart rate | 8 | 1.7% | Complete-case |
| LVEF | 12 | 2.6% | Complete-case |
| Serum creatinine | 15 | 3.2% | Complete-case |
| GDMT status | 0 | 0% | Complete-case |
| Prior hospitalization | 0 | 0% | Complete-case |

**Notes:**

- All missing data were <5% for each variable.
- Patterns of missingness were examined and considered missing at random.
- Analyses were
